# Exploring the Potential of Mesenchymal Stem Cell-Based Therapy in Mouse Models of Vascular Cognitive Impairment

**DOI:** 10.3390/ijms21155524

**Published:** 2020-08-01

**Authors:** Na Kyung Lee, Hyeongseop Kim, Jong Wook Chang, Hyemin Jang, Hunnyun Kim, Jehoon Yang, Jeyun Kim, Jeong Pyo Son, Duk L. Na

**Affiliations:** 1 School of Medicine, Sungkyunkwan University, 81 Irwon-ro, Gangnam-gu, Seoul 06351, Korea; nakyunglee@skku.edu; 2 Stem Cell & Regenerative Medicine Institute, Samsung Medical Center, 81 Irwon-ro, Gangnam-gu, Seoul 06351, Korea; hyeongseop09@gmail.com (H.K.); jongwook.chang@samsung.com (J.W.C.); hmjang57@gmail.com (H.J.); 3 Samsung Alzheimer Research Center, Samsung Medical Center, 81 Irwon-ro, Gangnam-gu, Seoul 06351, Korea; 4 Stem Cell Institute, ENCell Co. Ltd., Seoul 06072, Korea; 5 Department of Neurology, Sungkyunkwan University School of Medicine, Samsung Medical Center, 81 Irwon-ro, Gangnam-gu, Seoul 06351, Korea; 6 Laboratory Animal Research Center, Samsung Biomedical Research Institute, 81 Irwon-ro, Gangnam-gu, Seoul 06351, Korea; mdpkhn.kim@samsung.com (H.K.); jehoon815.yang@samsung.com (J.Y.); jeyun604.kim@sbri.co.kr (J.K.); 7 Laboratory Animal Center, Osong Medical Innovation Foundation, Cheongju 28160, Korea; jpyo.son@gmail.com; 8 Department of Health Sciences and Technology, SAIHST, Sungkyunkwan University, 81 Irwon-ro, Gangnam-gu, Seoul 06351, Korea; 9 Neuroscience Center, Samsung Medical Center, 81 Irwon-ro, Gangnam-gu, Seoul 06351, Korea

**Keywords:** Alzheimer’s disease, vascular cognitive impairment, heterogeneity, mesenchymal stem cell, therapeutic

## Abstract

Closely linked to Alzheimer’s disease (AD), the pathological spectrum of vascular cognitive impairment (VCI) is known to be wide and complex. Considering that multiple instead of a single targeting approach is considered a treatment option for such complicated diseases, the multifaceted aspects of mesenchymal stem cells (MSCs) make them a suitable candidate to tackle the heterogeneity of VCI. MSCs were delivered via the intracerebroventricular (ICV) route in mice that were subjected to VCI by carotid artery stenosis. VCI was induced in C57BL6/J mice wild type (C57VCI) mice by applying a combination of ameroid constrictors and microcoils, while ameroid constrictors alone were bilaterally applied to 5xFAD (transgenic AD mouse model) mice (5xVCI). Compared to the controls (minimal essential medium (MEM)-injected C57VCI mice), changes in spatial working memory were not noted in the MSC-injected C57VCI mice, and unexpectedly, the mortality rate was higher. In contrast, compared to the MEM-injected 5xVCI mice, mortality was not observed, and the spatial working memory was also improved in MSC-injected 5xVCI mice. Disease progression of the VCI-induced mice seems to be affected by the method of carotid artery stenosis and due to this heterogeneity, various factors must be considered to maximize the therapeutic benefits exerted by MSCs. Factors, such as the optimal MSC injection time point, cell concentration, sacrifice time point, and immunogenicity of the transplanted cells, must all be adequately addressed so that MSCs can be appropriately and effectively used as a treatment option for VCI.

## 1. Introduction

Following Alzheimer’s disease (AD), vascular cognitive impairment (VCI) is a highly prevalent form of dementia among the aging population. VCI is an umbrella term that encompasses VCI-no dementia to vascular dementia (VaD) [1]. Many groups support the notion that a considerable overlap exists between cerebrovascular disease and AD [2,3]. While such coexistence is difficult to observe naturally in animal models, this overlap is commonly observed clinically in the aged [4]. Similar to AD, the etiology of VCI is known to be complex and heterogeneous [5]. Chronic hypoperfusion is considered to be one of the major pathogenic mechanisms underlying VCI [6,7,8]. Rodent models of chronic hypoperfusion that mimic features of VCI have been proposed by numerous groups [1,9]. Like many animal models for human diseases, a single rodent model that recapitulates the entire complicated spectrum of VCI is not available. However, by studying these animal models, researchers discovered that the pathogenic mechanisms and metabolic pathways underlying VCI seem to be interconnected [10]. For example, chronic hypoperfusion or ischemia is reported to initiate a cascade of events starting off with oxidative stress, which subsequently leads to neuroinflammation, neuronal atrophy, and finally cognitive deficits [10].

The bilateral common carotid artery stenosis method that involves the symmetric application of microcoils to the bilateral common carotid arteries (CCAs) has been widely used among researchers experimenting on VCI. Another common method to generate chronic hypoperfusion is the two-vessel occlusion (2VO) method that involves ligation of CCAs using silk sutures or micro clamps [11,12]. Depending on the objective of the study, arteries are temporarily ligated, and reperfusion is performed after a certain time point [13]. Previously, a group proposed a novel method to replicate major aspects of human VCI, such as cerebral infarcts and memory impairment, via asymmetric application of ameroid constrictors (right CCA) and microcoils (left CCA) [14]. Recently, we presented findings that when this model is applied to C57BL6/J and a transgenic Alzheimer’s disease model (5x familial Alzheimer’s disease; 5xFAD), the clinical heterogeneity of VCI is depicted [15].

Currently, there are no treatments that can permanently alter the clinical course of both AD and VCI. Since AD pathogenesis consists of multiple mechanisms [16], there is a consensus that a single target approach is not effective and that multi-target drugs can be a potential option to target the diverse aspects of the disease [17,18]. Equally, if not more complex, therapeutics for VCI will have to target both neuronal insult and the cerebrovascular pathology itself to obtain cognitive benefits and functional recovery [19,20]. Mesenchymal stem cells (MSC) have risen as a promising therapeutic candidate for diseases with complicated underlying pathologies, such as neurodegenerative diseases. MSCs retain promising features, such as the ability to reduce amyloid beta (Aβ) plaques [21], elevate endogenous neurogenesis [22], and upregulate proteasome activity [23]. Such wide-ranging activities are possible due to the secretion of cytokines or the paracrine activity of MSCs [24]. Other than neurodegenerative diseases, the immunosuppressive and immunomodulatory abilities of MSCs in bacterial infections have generated considerable recent interest [25]. For example, it has been proposed that MSCs can modulate the host immune response and generate antibacterial responses to tackle the bacterial cells [25]. Compared to VaD, numerous clinical [26] and preclinical studies [27] have been carried out to assess the safety and efficacy of MSCs in AD stem cell therapy. Very few studies have assessed the efficacy of bone marrow-derived MSCs in rodent models where chronic hypoperfusion was induced by the 2VO approach [28]. According to the existing literature, a very limited number of studies, if not none, have examined the potential of MSC therapy in rodent models where VCI has been induced via the combination of ameroid constrictors and microcoils or just ameroid constrictors alone. In addition, the potential of MSC therapy in mixed cases of AD and VCI is worth being explored considering their additive interaction on cognition [2,29,30,31,32,33,34] and the more valuable role of multi-target drugs in these cases.

The main objective of the present study was to investigate on the feasibility and efficacy of human MSC transplants as a potential treatment for VCI. VCI was induced by utilizing previously reported methods that involve a combination of ameroid constrictors and microcoils [14] or ameroid constrictors alone [35]. MSC injections were carried out using not only wild type (C57BL6/J) but also using a transgenic AD mouse model (5xFAD). Here, 5xFAD mice were subjected to VCI to assess the potential of MSCs being used as a therapeutic option for models where AD and vascular pathologies coexist.

## 2. Results

### 2.1. Experiment #1: Unexpectedly, the Survival of C57VCI Mice Was Decreased Following Transplantation of Human MSCs

**Figure 1:**
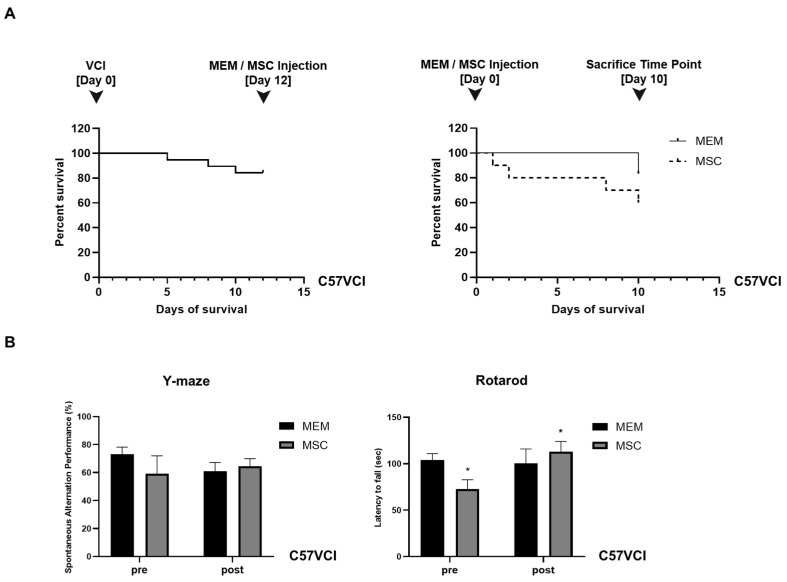
Assessment of changes in survival and behavioral performance following MSC injection for experiment #1. (**A**) Kaplan Meier survival curves drawn from (left graph) the time of VCI surgery (day 0; combination of ameroid constrictors and microcoils applied bilaterally to the CCAs of C57BL6/J mice to generate C57VCI mice) to MEM/MSC injections (day 12) and (right graph) also from the point of MEM/MSC injection (day 0) to the sacrifice time point (day 10). The dotted broken line indicates the survival results for the MSC group. (**B**) Behavioral tests performed to assess changes in spatial working memory (Y maze, top graph) and motor coordination (Rotarod, bottom graph) before (pre) and after (post) MEM/MSC injections to the C57VCI mice. * *p* < 0.05 vs. MSC (pre); mean ± S.E.M.

Human MSCs (1 × 10^5^/2 µL) were injected bilaterally into the lateral ventricles of C57VCI mice, 12 days after performing the VCI surgery (ameroid constrictor:right CCA, microcoil:left CCA). Based on the Kaplan Meier survival analysis, the survival rate of C57VCI mice on the day of MEM or MSC injection (12 days after inducing VCI) was 84.2% (Figure 1A, left graph). At the sacrifice time point (10 days after MEM/MSC injection), the survival rate of C57VCI mice after performing MEM injections was 83.3%, while contrary to our expectation, the survival rate after MSC injection was 60% (Figure 1A, right graph). Three C57VCI mice died at days 1, 2, and 8, respectively, after receiving MSC injections. Two mice died while acquiring the last MR images (MEM: *n* = 1, MSC: *n* = 1) for week 3.

### 2.2. Experiment #1: Spatial Working Memory and Motor Coordination Were Not Affected in C57VCI Mice after Transplantation of Human MSCs

According to the Y-maze results, a statistically significant difference in spatial working memory or spontaneous alternating performance (SAP)% was not observed when comparing the MEM and MSC groups (Figure 1B, left graph). Prior to MEM/MSC administration, the SAP% of the MEM and MSC groups were as follows: 73.0 ± 5.2% and 59.3 ± 12.7%. While the SAP% was lower for the MSC group, a statistically significant difference did not exist in comparison to the MEM group. After MEM or MSC administration, the SAP% of the MEM group dropped from 73.0 ± 5.2% to 61.0 ± 6.2% while the SAP% of the MSC group improved from 59.3 ± 12.7% to 64.5 ± 5.5%. Statistical significance was not noted when comparing the pre and post SAP% results separately for each of the groups.

Like the Y-maze results, according to the baseline evaluation (pre) of motor coordination, the MSC group exhibited shorter latency to fall (72.5 ± 10.3 s) in comparison to the MEM group (104.1 ± 6.8 s) (Figure 1B, right graph) and this difference was statistically significant (* *p < 0.05*). Interestingly, the latency to fall was higher and a statistically significant difference existed when comparing the pre and post (after MEM/MSC injection) behavioral results of the MSC group: (post) 113.1 ± 11 s. After MEM injection, the latency to fall for the MEM group was unchanged: 100.3 ± 15.7 s. When comparing the post rotarod results of the MEM and MSC groups, a statistically significant difference was not noted.

### 2.3. Experiment #1: C57VCI Mice Display Heterogeneity in Pathological Manifestation

**Figure 2:**
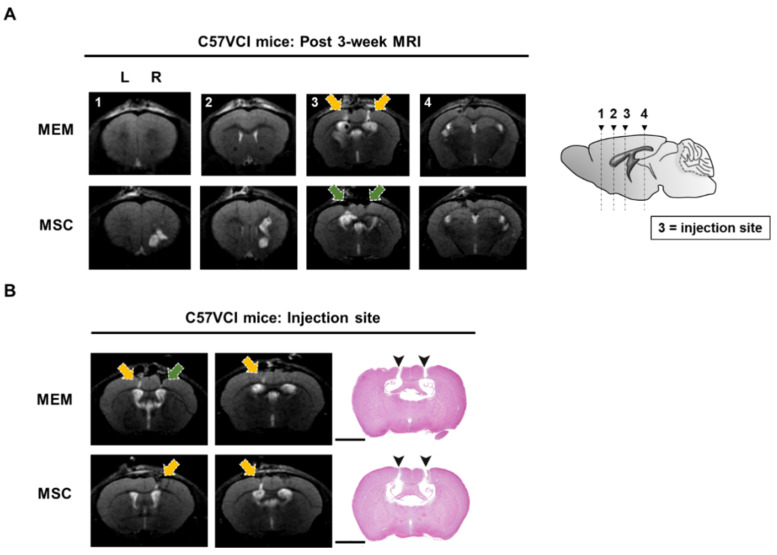
Identification of the injection point via MR image and histological stains. (**A**) Representative T2 weighted MR images acquired at post 3 weeks from C57VCI mice from the MEM and MSC groups. L indicates left and R indicates the right hemisphere of the mouse brain. The yellow solid arrow indicates hyperintense signals that appear as a vertical streak from the cortex to the hippocampus. The green solid arrow indicates minor damage to the cortex due to the penetration of the mouse parenchyma by the Hamilton syringe. The location of each of the coronal MR sections is illustrated by the sagittal section of the mouse brain (right): (1) forceps minor of corpus callosum (+1.70 mm anterior to bregma), (2) external capsule of the corpus callosum (+0.62 mm anterior to bregma), (3) the injection point (lateral ventricles) and the caudate putamen can be visualized (−0.22 mm posterior to bregma), and (4) the hippocampal fimbria and hippocampus can be detected (−1.82 mm posterior to the bregma). An infarct is detected in the left caudate putamen (section #3) of the representative C57VCI mouse from the MEM group and infarcts in the right anterior commissure (section #1) and corpus callosum (sections #2 and 3) are detected from the representative C57VCI mouse from the MSC group. (**B**) Similar histological sections that matched the MRI slices where hypointense or hyperintense signals were identified were stained with hematoxylin and eosin (H&E). Puncture of the cortex (solid black arrowheads) via the insertion of the Hamilton syringe was exhibited from the H&E stains. Scale bar = 2 mm.

As reported previously [14], the heterogeneous disease progression of the C57VCI mice was consistently observed from the current study. Out of the 10 C57VCI mice that received MSC injections, only 2 mice displayed signs of cerebral infarct at the sacrifice time point. From the two MSC-injected C57VCI mice, cerebral infarcts were identified in both the left (microcoil) and right (ameroid constrictor) hemispheres of one mouse while infarcts were only visualized in the right hemisphere of the second mouse. Moreover, only two of the six C57VCI mice that received MEM injections also showed signs of cerebral infarct. From the two MEM-injected C57VCI mice, infarcts appeared only in the left hemisphere for one mouse and only the right hemisphere for the second mouse. The infarcts for both the MEM and MSC-injected mice were detected not at varying time points but at post 3 weeks. According to the magnetic resonance (MR) images, infarcts developed in the anterior commissure, corpus callosum, caudate putamen, and the hippocampal fimbria (Figure 2A). The injection sites were identified as damage to the cortex (green solid arrow) or as vertical streaks with hyperintense signals (yellow solid arrow) from the MR images (Figure 2).

### 2.4. Experiment #1: Cell Aggregates Are Identified from Several C57VCI Mice that Received MSC Injections

**Figure 3:**
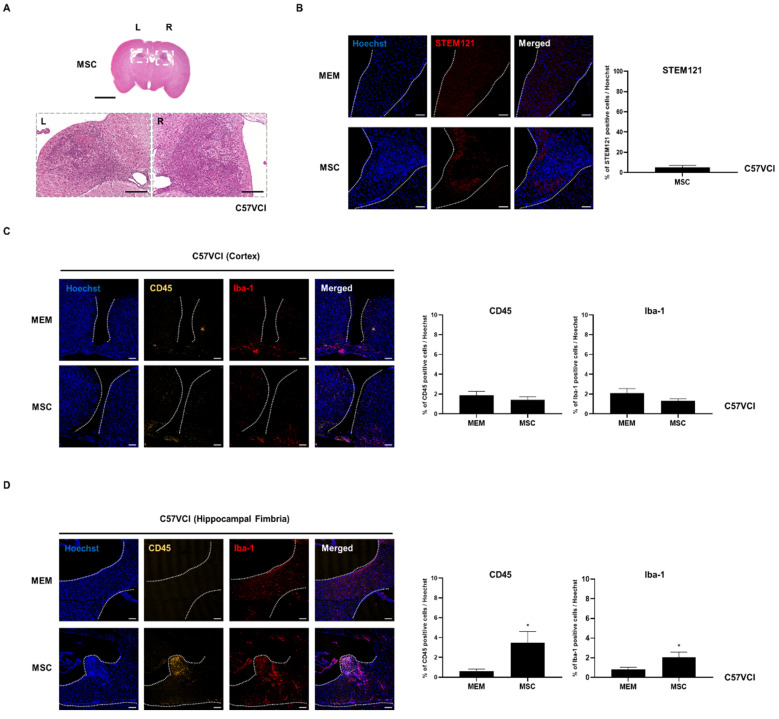
Cell aggregates identified from C57VCI mice that received MSC injections. (**A**) Based on the H&E stains, an accumulation of pyknotic cells was identified from the hippocampal fimbria of C57VCI mice from the MSC group (*n* = 6). L indicates left and R indicates the right hemisphere of the mouse brain. Scale bar = 2 mm (whole brain, top), 200 µm (bottom). (**B**) Immunohistochemical (IHC) staining using the STEM121 marker indicated that no human MSCs are present in the hippocampal fimbria (region demarcated with broken white lines) of the MEM group while a very small percentage of human MSCs is present in the cell aggregate of the MSC group. Scale bar = 100 µm. (**C**) CD45 and Iba-1 markers were used to detect the presence of leukocytes and microglia/macrophage, respectively. In the damaged area of the cortex (the gap in between the broken white lines displays the site of Hamilton syringe injection), MEM (*n* = 5) and MSC-injected C57VCI mice (*n* = 6) did not display significant differences in both CD45 and Iba-1 expression levels. Scale bar = 100 µm. (**D**) Compared to the MEM group, statistically significant differences in both CD45 and Iba-1 expression levels of the MSC group were observed in the hippocampal fimbria (region demarcated by broken white lines). * *p* < 0.05 vs. MEM; mean ± S.E.M. Scale bar = 100 µm.

Interestingly, from three of the six C57VCI mice (excluding the one mouse that died while acquiring MR images on the last day) that received bilateral injections of MSCs into the LV, cell aggregates were identified from the hippocampal fimbria (Figure 3A). Based on the H&E stains, the aggregates were densely populated with cells displaying pyknotic nuclei. According to immunohistochemistry (IHC), a small percentage of human cytoplasmic protein (STEM121)-positive human cells (5.1 ± 1.8%) or persisting human MSCs was identified from the C57VCI group that received MSC injections (Figure 3B). As expected, no STEM121-positive signals were observed from the C57VCI-MEM group. Other than STEM121-positive cells, the expressions of immune (leuckocyte common antigen; CD45) and inflammatory cells (ionized calcium-binding adapter molecule 1; Iba-1) were also investigated in the damaged area of the cortex caused by the insertion of a Hamilton syringe and also the hippocampal fimbria, where cell aggregates were identified from several C57VCI mice (Figure 3C,D). Statistically significant differences in both CD45 (MEM: 1.9 ± 0.4% vs. MSC: 1.4 ± 0.3%) and Iba-1 (MEM: 2.1 ± 0.5% vs. MSC: 1.3 ± 0.2%) expression levels were not discernible in the damaged area of the cortex (gap in between demarcated broken white lines) (Figure 3B). In contrast to the cortex, a statistically significant difference in both CD45 (MEM: 0.6 ± 0.2% vs. MSC: 3.5 ± 1.1%) and Iba-1 (MEM: 0.8 ± 0.2% vs. MSC: 2.0 ± 0.5%) expression levels existed between the two groups in the hippocampal fimbria. Compared to that of the MEM-injected C57VCI mice, the CD45 and Iba-1 expression levels of the MSC-injected C57VCI mice were greater by 5.5- and 2.5-fold, respectively (Figure 3D).

Considering that MSCs were intended to be injected into the bilateral LVs of the C57VCI mice, it was questionable as to why cell aggregates were identified from the hippocampal fimbria of three C57VCI mice. Thus, additional analysis of MR images and coronal serial sections of the mouse brain past the lateral ventricles was performed. From the three C57VCI mice that exhibited cell aggregates in the hippocampal fimbria, a vertical needle streak was identified in the hippocampus as a hypointense signal from the T2 weighted MR images (Figure S1). Hypointense signals were also identified from the ventral part of the hippocampus and the hippocampal fimbria. The vertical needle streak was confirmed from the H&E stains (broken white line). The ventral part of the hippocampus was lined with pyknotic cells (Figure S1).

### 2.5. Experiment #1: Neuronal Density and White Matter Tracts of C57VCI Mice Are Unaffected after MSC Injection

**Figure 4:**
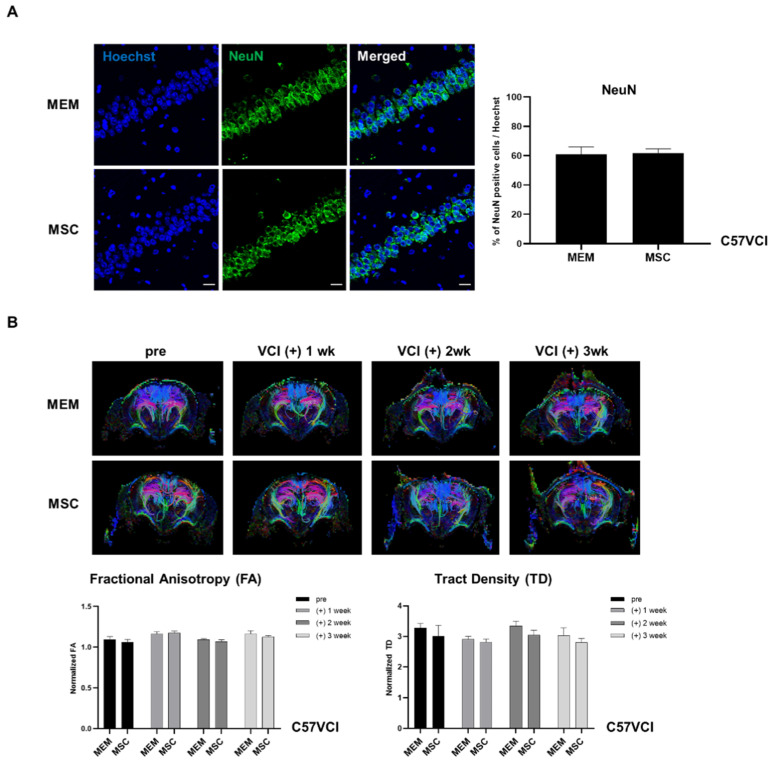
Evaluation of changes in neuronal density and white matter tracts after MSC injection. (**A**) IHC stains displaying expressions of hippocampal neuronal cells (green) using the mature neuron marker, NeuN, in both the MEM (*n* = 5) and MSC (*n* = 6) groups. Scale bar = 20 µm. (**B**) Directional color-coded fractional anisotropy (FA) maps (acquired at varying time points: pre, post 1 week: (+) 1 wk, post 2 week: (+) 2 wk, and post 3 week: (+) 3 wk) exhibiting the tractography of representative animals from the MEM and MSC groups. Selective DTI parameters: FA and tract density (TD) are normalized by dividing the value of the corpus callosum from the value of the whole brain.

**Figure 5:**
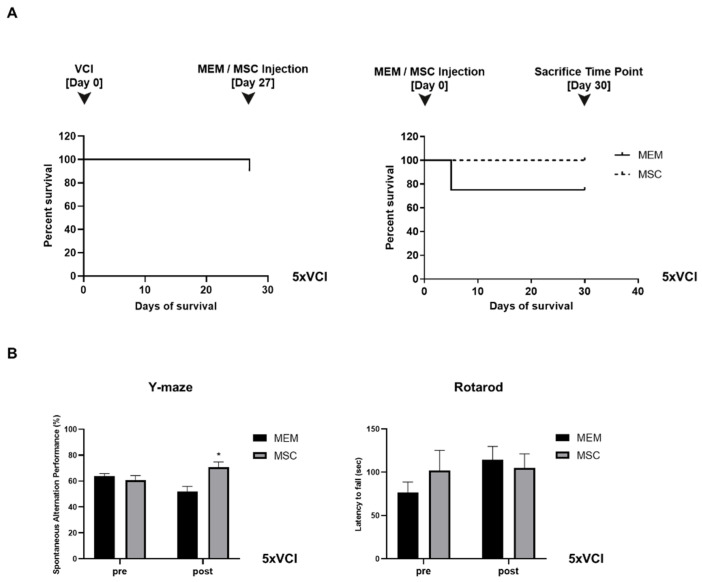
Assessment of changes in survival and behavioral performance following MSC injection for experiment #2. (**A**) Timeline for experiment #2. (**A**) Kaplan Meier survival curves drawn from (left graph) the time of VCI surgery (day 0; ameroid constrictors bilaterally applied to the CCAs of 5xFAD mice to generate 5xVCI mice) to MEM/MSC injections (day 27) and (right graph) also from the point of MEM/MSC injection (day 0) to the sacrifice time point (day 30). The dotted broken line indicates the survival results for the MSC group. (**B**) Behavioral tests performed to assess changes in spatial working memory (Y maze, top graph) and motor coordination (Rotarod, bottom graph) before (pre) and after (post) MEM/MSC injections to the C57VCI mice. * *p* < 0.05 vs. MEM (post); mean ± S.E.M.

Since hippocampal neuronal loss is another hallmark of C57VCI mice along with cerebral infarcts [14], hippocampal neuronal density was examined by using the mature neuronal nuclear protein, NeuN. A statistically significant difference did not exist when comparing the hippocampal neuronal density between the C57VCI-MEM (60.9 ± 5.1%) and MSC (61.9 ± 2.9%) groups (Figure 4A). Compared to the C57VCI mice that received MEM injections, statistically significant differences in diffusion tensor imaging (DTI) parameters were not observed in the corpus callosum (CC) of the MSC-injected group (Figure 5B). The normalized fractional anistropy (FA) and tract density (TD) values (normalized: entire CC including infarct/whole brain

DTI indices were quantitated (Figure 4B) by analyzing the diffused weighted images acquired at varying time points: pre, post 1 week, 2 week, and 3 weeks. Normalized FA values for the C57VCI-MEM and MSC groups were as follows: (1) MEM:1.10 ± 0.04 (pre), 1.26 ± 0.03 (post 1 week), 1.09 ± 0.01 (post 2 week), and 1.16 ± 0.04 (post 3 week); (2) MSC:1.06 ± 0.03 (pre), 1.27 ± 0.02 (post 1 week), 1.07 ± 0.02 (post 2 week), and 1.13 ± 0.02 (post 3 week). Normalized TD values for the C57VCI - MEM and MSC groups were as follows: (1) MEM:3.28 ± 0.15 (pre), 2.92 ± 0.09 (post 1 week), 3.34 ± 0.16 (post 2 week), and 3.04 ± 0.24 (post 3 week); (2) MSC:3.00 ± 0.36 (pre), 2.82 ± 0.10 (post 1 week), 3.05 ± 0.15 (post 2 week), and 2.82 ± 0.13 (post 3 week). While the normalized TD values were slightly reduced for the MSC group, especially at post 2 and 3 weeks, a statistically significant difference did not exist compared to the MEM-injected group.

### 2.6. Experiment #2: Mortality Was Not Observed from the MSC-Injected 5xVCI Mice

First, 5xFAD mice received MEM or MSC injections 4 weeks or a month after inducing VCI via bilateral application of ameroid constrictors (5xVCI). Mice were sacrificed 4 weeks or a month later after the injection. Based on the Kaplan Meier survival analysis, the survival rate of 5xVCI mice on the day of MEM or MSC injection (27 days after inducing VCI) was 90% (Figure 5A, left graph). At the sacrifice time point (30 days after MEM/MSC injection), the survival rate of 5xVCI mice after performing MEM or MSC injections was 75% and 100%, respectively (Figure 5A, right graph). One mouse died after receiving MEM injections at day 5.

### 2.7. Spatial Working Memory is Enhanced Following Injections of MSCs in 5xVCI Mice

According to the Y-maze results, prior to MEM/MSC administration (pre), the spatial working memory or spontaneous alternating performance (SAP) % of the MEM and MSC groups were as follows: 63.6 ± 2.2% and 52.0 ± 3.9%, respectively (Figure 5B). When the SAP% was measured a month after performing the MEM/MSC injections, a statistically significant difference was noted between the MEM and MSC groups (Figure 5B, left graph). Compared to the MEM group (52.0 ± 3.9%), the SAP% of the MSC group (70.7 ± 4.1%) was greater by 1.4-fold (^*^p < 0.05) (Figure 5B, left graph). Statistical significance was not observed when comparing the pre and post SAP% of the MEM and MSC groups separately.

Like the C57VCI mice from experiment #1, the motor coordination of 5xVCI mice was not affected following injections of MSCs into the lateral ventricle (Figure 5B, right graph). Prior to injecting MEM or MSCs, the MEM group exhibited a shorter latency to fall (76.5 ± 12.0 s) in comparison to the MSC group (101.9 ± 23.2 s). Differences were not statistically significant. Compared to the baseline evaluation (pre), the latency to fall was longer for the MEM group (114.1 ± 15.5 s). Moreover, according to the post rotarod results, the latency to fall for the MSC group (104.7 ± 16.4 s) was shorter in comparison to that of the MEM group (Figure 5B, right graph). Differences were not statistically significant.

### 2.8. 5xVCI Mice Display Subtle Changes in Disease Progression

**Figure 6:**
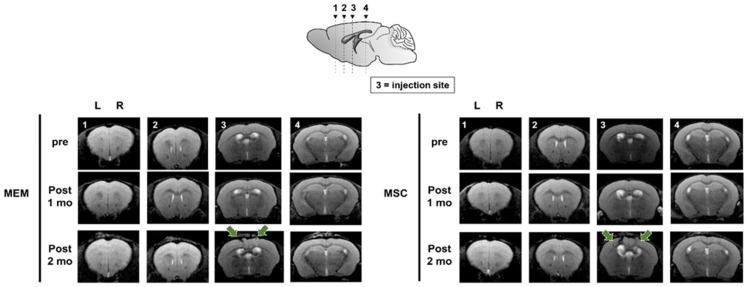
MR images of the 5xVCI mice acquired at varying time points. T2 weighted MR images from representative animals from the MEM (left) and MSC (right) groups are shown. MR images were acquired at pre, post 1 mo (a month following VCI surgery), and post 2 mo (2 months following VCI surgery, 1 month after MEM or MSC injections). Solid green arrows signify slight damage to the cortex generated by penetration of the Hamilton syringe. Signs of cerebral infarcts are not noted from the MR images at all time points for both the MEM and MSC groups. L indicates left and R indicates the right hemisphere of the mouse brain. The location of each of the coronal MR sections is illustrated by the sagittal section of the mouse brain (top): (1) forceps minor of the corpus callosum (+1.70 mm anterior to bregma), (2) external capsule of the corpus callosum (+0.62 mm anterior to bregma), (3) the injection point (lateral ventricles) and the caudate putamen can be visualized (−0.22 mm posterior to bregma), and (4) the hippocampal fimbria and hippocampus can be detected (−1.82 mm posterior to bregma).

**Figure 7:**
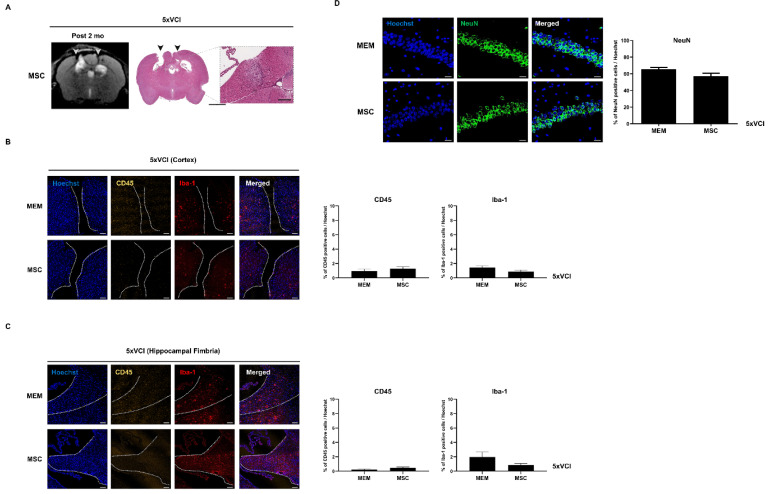
Pathological manifestation of the 5xVCI mice following MEM/MSC injection. (**A**) T2 weighted image of a representative animal from the 5xVCI-MSC group is shown on the left. Damage to the cortex caused by the penetration of the Hamilton syringe is indicated with solid gray arrowheads. According to an H&E stain of a section equivalent to the MR image slice, a faint round developing infarct can be detected from the hippocampal fimbria (*n* = 2 of 5). Signs of the infarct could not be noted from the MR image. Signs of penetration into the cortex via the Hamilton syringe are indicated by solid black arrowheads. Based on IHC staining, high populations of Iba-1-positive microglia/macrophage cells (indicated in red) are visualized from the site of the developing infarct. Scale bars (left to right) = 2 mm (whole brain), 200 µm, 20 µm (IHC stain). (**B**) CD45 and Iba-1 markers are used to detect the presence of leukocytes and microglia/macrophage, respectively. In the damaged area of the cortex (gap in between broken white lines display the site of Hamilton syringe injection), the expression of both CD45 and Iba-1 markers is extremely low for both groups and a statistically significant difference does not exist between the MEM (*n* = 3) and MSC (*n* = 5) groups. Scale bar = 100 µm. (**C**) In the hippocampal fimbria (region demarcated by broken white lines), notable differences in CD45 and Iba-1 expression levels are not observed between the 2 groups. Scale bar = 100 µm. (**D**) Based on NeuN immunostaining (green), there is no remarkable difference in hippocampal neuronal density of the MEM and MSC groups. Scale bar = 20 µm.

T2 weighted MR images were acquired prior to VCI surgery (pre), a month after the surgery (post 1 mo), and another month later following MEM/MSC injection (post 2 mo) (Figure 6). For both the MEM and MSC groups, hyperintense signals or signs of infarcts were not detected in areas, such as the caudate putamen, corpus callosum, and hippocampus (Figure 6). From the post 2 mo MR images (taken a month after carrying out MEM/MSC injections), slight damage to the cortex was evident due to the insertion and withdrawal of the Hamilton syringe during injection (solid green arrows; Figure 6). MR images were corroborated by histology (H&E stains). Interestingly, out of the 5 5xVCI mice from the MSC group, a small developing infarct was identified from the hippocampal fimbria of two mice. Signs of this infarct were not visible from the MR images (Figure 7A). Based on the H&E stains, cells with a foamy appearance or macrophages [36] were visible from the infarct site. IHC was conducted subsequently to examine for the presence of inflammatory cells. Strong expressions of the Iba-1 antibody, which is widely known as a microglia and macrophage marker [37], was visible from the infarct site (Figure 7A). Subsequently, the expression of CD45 and Iba-1-positive cells was closely examined in the damaged area of the cortex (caused by insertion of the Hamilton syringe; gap in between demarcated broken white lines) and the hippocampal fimbria of both MEM and MSC-injected 5xVCI mice. Overall, in the cortex, the CD45 and Iba-1 expression levels were very low in both the MEM (CD45: 0.2 ± 0.1%, Iba-1: 1.4 ± 0.2%) and MSC (CD45: 0.5 ± 0.1%, Iba-1: 0.9 ± 0.2%) groups and a remarkable difference did not exist between the two groups (Figure 7B). Moreover, a significant difference in CD45 expression levels was also not evident in the hippocampal fimbria (region demarcated by a broken white line) of both MEM and MSC-injected mice (Figure 7C). Although a slight decrease in the Iba-1 expression levels was noted in the hippocampal fimbria of MSC-injected 5xVCI mice (0.8 ± 0.2%), differences were not statistically significant when compared to that of the MEM group (2.0 ± 0.7%) (Figure 7D). Along with the detection of these small developing infarcts, the NeuN antibody was used to observe alterations in hippocampal neuronal density. Compared to the MEM group (65.6 ± 1.9%), a slight reduction in the expression of NeuN-positive mature neurons was observed from the MSC group (57.3 ± 3.4%), but the difference was not statistically significant (Figure 7B).

### 2.9. Amyloid Beta Levels of 5xVCI Mice Are Altered in the Thalamus Following MSC Injection

**Figure 8:**
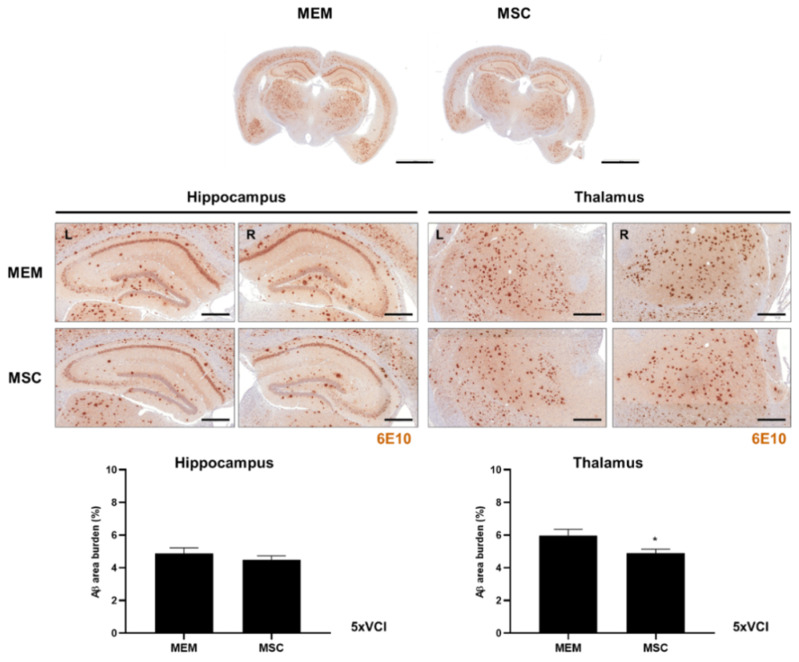
Assessment of amyloid deposition of 5xVCI mice following MEM/MSC injections. Expression of the Aβ protein (6E10 antibody; brown) in the hippocampus and thalamus of 5xVCI-MEM and MSC groups were evaluated via IHC staining. L indicates left and R indicates the right hemisphere of the mouse brain. Scale bars = 2 mm (whole brain), 400 µm. * *p* < 0.05 vs. MEM; mean ± S.E.M.

The 6E10 amyloid beta (Aβ) 1-42 antibody was utilized to investigate the changes in Aβ levels in the hippocampus and thalamus of 5xVCI following MEM / MSC injection. The percentage of Aβ burden for the hippocampus and thalamus was quantitated by averaging the results obtained from both hemispheres. As observed from the IHC stains using the NeuN marker, statistically significant differences were not noted in the neuronal densities of the MEM (65.6 ± 1.9%) and MSC (57.3 ± 3.4%) groups (Figure 7B). Similarly, significant differences in amyloid burden (%) levels were not noted based on gross observation of the left and right hippocampi of 5xVCI mice that received either MEM or MSC injections (Figure 8). Compared to the MEM group (4.9 ± 0.4%), the MSC group (4.5 ± 0.3%) displayed a slight increase in the amyloid burden level, but the differences were not statistically significant (Figure 8). In the thalamus, the MSC group (4.9 ± 0.2%) exhibited lower Aβ levels than the MEM group (6.0 ± 0.4%) and differences were statistically significant.

## 3. Discussion

To the best of our knowledge, this is the first study that has investigated the feasibility and efficacy of ICV-delivered human MSCs in mouse models that were subjected to vascular cognitive impairment (VCI) surgery via implantation of a combination of ameroid constrictors and microcoils or ameroid constrictors alone.

As we reported recently [15], the heterogeneous disease progression of the C57VCI mice that underwent asymmetric vascular compromise was observed again from this current study (experiment #1), which was different from those of 5xVCI mice (experiment #2). Cerebral infarcts were only identified from a few of the C57BL6/J mice (both MEM and MSC-injected groups) that were subjected to VCI. For example, signs of cerebral infarcts were observed from only 2 out of 6 C57VCI that received MEM injections and 2 out of 10 C57VCI mice that received MSC injections. As shown from the MR images in Figure 2, the sizes and location of infarcts in terms of the anatomical site and the affected hemisphere varied widely among the mice (both MEM and MSC groups) that exhibited cerebral infarcts. Such results highlighted the heterogeneous disease progression of the C57VCI mice regardless of MEM or MSC injection. In contrast, the 5xVCI mice (experiment #2) did not show signs of distinct developed cerebral infarcts even on both MR images and H&E stains. Instead, there were two out of five MSC-injected 5xVCI mice where small developing micro-infarcts were detected from the hippocampal fimbria. Such differences in pathological manifestation could have occurred again due to differences in the method of vascular compromise explained as follows.

**Figure 9:**
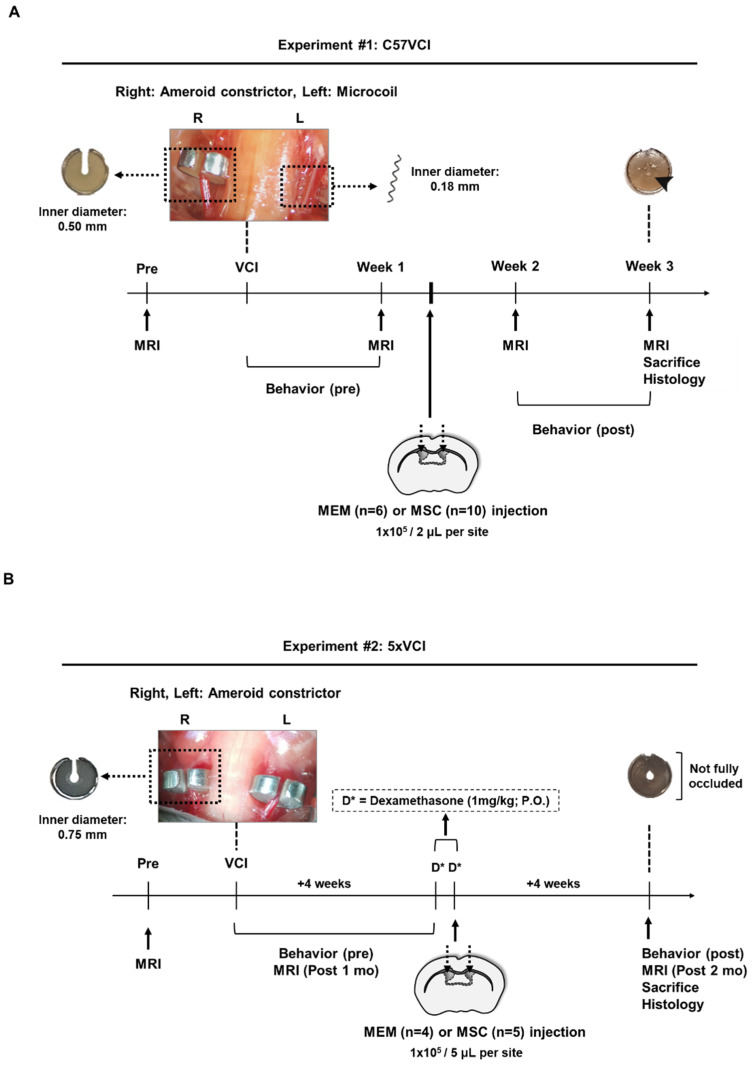
Study design schematic. (**A**) Timeline for experiment #1 (C57VCI). L indicates left and R indicates the right hemisphere of the mouse brain. A picture taken during the surgical process (upper left inset). An ameroid constrictor with an inner diameter of 0.5 mm and microcoil with an inner diameter of 0.18 mm are applied to the right and left common carotid arteries (CCAs), respectively. The lumen or inner material of the ameroid constrictor gradually narrows and by 3 weeks reaches near occlusion (indicated as solid black arrowhead). The dotted arrows on the coronal section of the mouse brain indicate the location where MEM or MSCs have been injected. (**B**) Timeline for experiment #2 (5xVCI). A picture taken during the surgical process (upper left inset). Ameroid constrictors with an inner diameter of 0.75 mm are bilaterally wrapped around the CCAs. The dotted arrows on the coronal section of the mouse brain indicate the location where MEM or MSCs have been injected. The lumen or inner material of the ameroid constrictor gradually narrows, and by 8 weeks or 2 months after surgery, the opening is closed but complete occlusion is not achieved. D* = dexamethasone (oral administration, 1 mg/kg).

Whereas for the C57VCI mice (ameroid constrictor with an inner diameter of 0.5 mm unilaterally applied to right CCA), ameroid constrictors with an inner diameter of 0.75 mm were applied bilaterally to the CCAs of 5xVCI mice. As shown in Figure 9, while the ameroid constrictors for the C57VCI mice reached near occlusion at the sacrifice time point, the ameroid constrictors of the 5xVCI were far from occlusion at the time of sacrifice, although narrowing of the lumen was evident. It has been reported that occlusion of ameroid constrictors has not yet been observed past 1 month [38]. Even though the 5xVCI mice were sacrificed past 1 month, at 2 months, the ameroid constrictors (inner diameter of 0.75 mm) still had not reached complete occlusion. Such results indicated that the blood flow of the 5xVCI mice was not severely impeded to generate multiple infarcts. This could have occurred since the inner diameters of the ameroid constrictors applied to the 5xVCI mice (0.75 mm) were larger than those applied to C57VCI mice (0.5 mm). Similarly, another group that utilized the symmetric approach by ligating the arteries of Wistar rats using silk sutures instead of ameroid constrictors detected no signs of cerebral infarcts up to 90 days [39].

The different method of vascular compromise seems to have affected not only the overall pathological manifestation but also the survival of the VCI-subjected mice. The low survival rate of C57VCI mice was similar to results from our previous study, where only 6 out of 17 C57VCI mice survived up to the 32-day endpoint [15]. Before carrying out MEM/MSC injections, the survival rates of C57VCI and 5xVCI mice were 84.2% and 90%, respectively. Thus, the survival of 5xVCI mice that received bilateral applications of ameroid constrictors was higher than that of the C57VCI mice that received an asymmetric application of ameroid constrictors and microcoils. However, it is important to note that MEM/MSC injections were performed 12 and 27 days after inducing VCI for the C57VCI and 5xVCI mice, respectively. This suggests that if MEM/MSC injections were performed at the equivalent time point of 5xVCI mice (27 days after inducing VCI), the survival rate of the C57VCI mice would have been much lower than 84.2%. If, oppositely, MEM/MSC injections were performed in 5xVCI mice 12 days instead of 30 days after injection, there is a high probability that the survival rate would have been 100% considering that death of one mouse was evident at day 27 (Figure 5A). If the MEM and MSC-injected C57VCI mice were observed up to 32 days like our previous study [15], there is a high possibility that the overall survival rate for both groups would have been lower.

Other than differences in vascular compromise, the cell concentration could have also factored in affecting the overall survival of the VCI-subjected mice. A former group injected 2 × 10^5^ MSCs suspended in 2 µL of Hank’s balanced salt solution (HBSS) bilaterally into the lateral ventricles of 6-month-old 5xFAD mice [40]. The authors proposed that therapeutic benefits were enhanced when MSCs were bilaterally injected into the lateral ventricles and not the bilateral hippocampi. Similarly, in experiment #1 of our study, 1 × 10^5^ MSCs suspended in 2 µL of phenol red free minimal essential medium alpha 1x (MEMα1x) were bilaterally injected into the LVs of C57VCI mice. Bilateral injection was performed so that MSCs were able to reach pathological lesions heterogeneously distributed in the VCI-subjected mice. The survival rate of the C57VCI mice that received MSC injections, however, was 60% while those that of the mice that received MEM injections was 83.3%. This could have again occurred due to the natural heterogeneous progression of the disease, but since a lower survival rate was identified following MSC and not MEM injection, we could not rule out the possibility that cell concentration could have been a contributing factor in increasing the mortality rate. For experiment #2, a more diluted concentration was used by increasing the suspension volume to 5 µL in the hope of increasing the survival rate and to achieve a more widespread distribution of MSCs in the mouse brain [41]. Additionally, 5xVCI mice received oral administrations of the corticosteroid, dexamethasone [42], prior to MEM/MSC injection. Although this administration was carried out to counteract immune responses generated by the human MSCs, whether the use of the corticosteroid affected the survival and disease progression of 5xFAD mice have not been investigated deeply in this study.

The presence of residual MSCs was not observed from both C57VCI and 5xVCI mice at 10 and 30 days after MSC injection, respectively. The cerebrospinal fluid (CSF) turnover for human and mice was reported to be around 1.8 and 4.8 h, respectively [43]. Thus, the CSF turnover of mice is approximately 2.7× faster than that observed from human subjects. Due to the rapid CSF flow, MSCs, regardless of the method of vascular compromise, could have been washed out of the circulatory system. We recently reported that the persistence of MSCs injected into the parenchyma (caudate putamen) was extremely low and barely detectable when sacrificed 7 days after injection [44], while for our study, C57VCI and 5xVCI mice were sacrificed 10 and 30 days after MEM/MSC injection, respectively. Thus, the time of sacrifice and the CSF flow provide possible explanations to the absence of residual MSCs in both C57VCI and 5xVCI mice. While MSC persistence was low following injection into the caudate putamen, we did, however, observe a high infiltration of immune cells at the injection site [44]. Similar observations were made in the hippocampal fimbria of three of the MSC-injected C57VCI mice. Although such observations were made due to inaccurate injections into the hippocampus (Supplementary Material Figure S1), signs of immune cells were not observed from any of the MEM-injected C57VCI mice. Such results suggest the immunogenicity of human MSCs. In addition to the xenogeneic origin of the MSCs, considering previous reports that the use of xenogeneic serum can induce immunogenicity of cells [45], the use of FBS in the culture of MSCs could have partly contributed to the elevated levels of CD45-positive leukocyte expression in the MSC-injected C57VCI mice. Although MSCs were washed in Dulbecco’s phosphate-buffered saline (DPBS) and suspended in serum-free phenol red free MEMα1x media, minute traces of FBS may still have been present since FBS was included in the media used to culture MSCs.

The hippocampal mature neuron (NeuN) densities were unchanged following MSC injection for both C57VCI and 5xVCI mice. It has been reported in the past that white matter lesions and hippocampal changes induce an impairment in working and reference memory [46]. For instance, in mice that underwent hypoperfusion, a correlation existed in white matter damage and selective spatial working memory impairment [47]. Based on the DTI-based tractography results of the C57VCI mice, significant differences in both the FA and TD indices were not observed from the MSC group in comparison to the MEM group for up to 3 weeks. This suggested that white matter damage was not clearly apparent from the C57VCI mice that received MSC injections, and thus, the hippocampal neuronal density remained unaltered overall. Although hippocampal neuronal loss has been reported to be a characteristic of the asymmetric vascular compromise model, clinically, a marked difference in the hippocampal neuronal count in the CA1 region did not exist between control subjects and VaD patients [48], which highlighted that neuronal loss is not a crucial and necessary pathophysiological mechanism for VaD to occur [20].

In experiment #1, a statistically significant difference in motor function was identified between the MEM and MSC groups at baseline (pre). The C57VCI mice underwent this assessment prior to receiving either MEM or MSC injections. As reported previously, heterogeneous disease progression or high individual variation was observed from C57BL6/J mice that were subjected to VCI [15]. Such occurrences seem to have been replicated in this present study. It has also been proposed that inducing chronic cerebral hypoperfusion in rodent models lead to deficits in motor function [49]. Due to the individual variation in the hemodynamics/disease progression of the VCI-subjected mice, which would have subsequently affected the motor function to different extents, may have accounted for the difference that existed between the two groups at baseline (pre). However, it is uncertain as to whether changes in the hemodynamics over time or the therapeutic effects of MSCs have generated the significant difference when comparing the pre and post motor function results of the MSC group. There have been reports that intracerebroventricular injections of MSCs in newborn rats with severe intraventricular hemorrhage improved the overall rotarod results [50].

It is noteworthy to mention the relationship between spatial working memory and neuronal density. It may be expected that an increase in spatial working memory would correlate with an increase in hippocampal neuronal density. One group, however, reported that although histological alterations in the hippocampus were not observed from mice that underwent chronic hypoperfusion (2VO), impairment in the spatial water maze task was discernible [51,52]. In our study, the spatial working memory or SAP% of MSC-injected 5xVCI mice was higher than that of the MEM-injected mice. When changes in hippocampal neuron densities were analyzed, a slight decrease in NeuN-positive cells was observed from MSC-injected 5xVCI; however, a statistically significant difference did not exist, thus signifying that a remarkable difference is not present between the MEM and MSC groups. Our results provide support to findings specifically from chronic hypoperfusion studies that a direct correlation between spatial working memory and hippocampal neuronal density may not always be present.

As reported previously, the 6E10 Aβ antibody used in this study specifically reacts towards not only amyloid plaques but also the hippocampal neuronal cells, where the Aβ proteins are also expressed [15]. The absence of prominent cerebral infarcts in the hippocampus provide a possible explanation as to why the neuronal count and thus the overall level of amyloid burden remained unchanged for both the MEM and MSC groups. Alternatively, the presence of hypoperfusion could have decreased the potential of MSCs in targeting AD pathologies. If the 5xVCI mice were sacrificed at a time point > 2 months, due to the progression of the disease, changes in amyloid deposition could have been more distinctly visible and apparent between the two groups. The overall amyloid burden of 5xVCI mice could have been altered if multiple instead of single injections of MSCS were carried out. It is worth noting that amyloid burden was reduced in the thalamus of MSC-injected 5xVCI mice. A previous group reported that the injection of brain-derived neurotrophic factor into the lateral ventricles increased the endogenous neurogenesis of adult rats [53]. Thus, although it has not been closely examined in this present study, paracrine factors secreted by the MSCs injected into the lateral ventricles could have played a role in ameliorating the amyloid burden of 5xVCI mice.

A limitation of this study is that two different mouse models with different experimental designs were incorporated into experiments #1 and #2, respectively. Since little work has been done previously to thoroughly examine the experimental conditions of performing MSC transplantations in mice that have been subjected to VCI via a combination of ameroid constrictors and microcoils, or ameroid constrictors alone, it was necessary to carry out a preliminary study to optimize the conditions. While 5xVCI mice closely resemble features seen in humans where the co-existence of AD and vascular pathologies are highly prevalent, this mouse model was not used for the preliminary study in that the co-occurrence of two different pathologies can serve as a confounder in optimizing the experimental conditions to perform MSC transplantations. Thus, the experimental design for the 5xVCI mice in experiment #2 was designed by referring to results obtained from experiment #1, where MEM or MSCs were injected into the LVs of C57VCI mice.

The optimal MSC injection time point, cell concentration, and sacrifice time point are factors that must be addressed and modified depending on the method of vascular compromise. One group previously reported that 3 days after inducing chronic hypoperfusion (2VO approach) in a rat model, bone marrow MSCs were injected via the tail vein [54]. The behavioral performance (Morris water maze) of the rats that received bone marrow MSCs was enhanced when evaluated 4 weeks after performing the injection. Compared to our study, behavioral assessment was performed a month after MSC injection, which was equivalent to our study. However, MSC injection was performed 3 days after inducing chronic hypoperfusion while in our study, it was 12 and 27 days for the C57VCI and 5xVCI mice, respectively. Again, this stresses how the injection time point must be optimized based on the disease progression of the respective VCI model. Lastly, if xenogeneic MSCs are used, the immune responses generated from these cells should also be taken into consideration.

## 4. Materials and Methods

### 4.1. Ethical Statement

This study was reviewed and approved (experiment #1: Approval number: 20170605001, Date: 5 June 2017; experiment #2: Approval number: 20200107003, Date: 7 January 2020) by the Institutional Animal Care and Use Committee (IACUC) of the Samsung Biomedical Research Institute (SBRI) at Samsung Medical Center (SMC). SBRI abides by the Institute of Laboratory Animal Resources (ILAR) guide and is an Association for Assessment and Accreditation of Laboratory Animal Care International (AAALAC International) accredited facility.

### 4.2. Experimental Animals and Study Design

The experimental timelines for experiment #1 and #2 are illustrated in Figure 9. C57BL6/J and 5xFAD mice (Jackson Laboratory, Bar Harbor, ME, USA) were maintained by mating a 5xFAD male mice with a C57BL6/J female mouse. Genotyping was carried out using tail snips from the offspring to separate transgenic and non-transgenic littermates. A total of 19 C57BL/6J (>3-month-old) mice were subjected to VCI for experiment #1 (Figure 9A). Twelve days after inducing VCI, *n* = 6 mice were randomly allocated to receive minimal essential medium (MEM) injections and the remaining *n* = 10 received MSC injections into the lateral ventricle, respectively. These mice were sacrificed 10 days after receiving MEM/MSC injections. Three mice died before performing MEM or MSC injections. Experiment #2 comprised of a total of 10 5× familial Alzheimer’s disease (5xFAD) transgenic AD mice (6 months old) (Figure 9B). A month after inducing VCI (4 weeks), mice were randomly allocated to receive MEM (*n* = 4) or MSC (*n* = 5) injections, respectively, into the lateral ventricle. All 9 mice received oral administrations of dexamethasone (1 mg/kg) for 2 days (day before MEM/MSC administration, and the day of the administration). These mice were sacrificed another month (4 weeks) after performing the MEM/MSC injections. The one mouse that died before receiving MEM or MSC injections died before receiving the oral administration of dexamethasone. All mice were fed ad libitum and were maintained in a 12-h light/12-h dark cycle.

For both experiments #1 and #2, a long-term cell tracer dye was not used to track the fate of the transplanted human MSCs. Through a recent study, we observed an extremely low persistence of human MSCs (unlabeled) at 7 days following transplantation of the cells into the caudate putamen (parenchyma) of wild type C57BL/6 mice [44]. Since the sacrifice time points for both experiment #1 and #2 surpassed the 7 days: 12 days and 30 days following MSC injection, respectively, we expected that residual human MSCs would be barely detectable from the MSC-injected mice in both experiments. Second, the rapid CSF turnover in mice [43] would have further facilitated the washout of the injected human MSCs from the circulatory system. Thus, it would have been difficult to track the fate of dye-labeled MSCs up to 30 or even 12 days. Reporter genes or labeling stem cells with iron oxide nanoparticles could have been incorporated into the study. However, a previous group reported that 4 weeks following transplantation of iron-oxide-labeled syngeneic rat MSCs into the hearts of rat myocardial infarction models, very few or no 4′,6-diamidino-2-phenylindole (DAPI)-positive stem cells were identified from the heart tissue [55]. Iron-positive signals that were identified at 4 weeks originated from CD68-positive macrophages and these macrophages were thought to have phagocytosed the iron particles. Thus, the authors concluded that iron signals visualized from MR images may not necessarily and reliably indicate the presence of stem cells in the target tissue.

### 4.3. VCI Surgery

Previously reported methods were referred to subject mice to VCI [15]. During surgery, isoflurane (Hana Pharmaceutical Co., Ltd., Seoul, Korea) was maintained at 1.5%–2%. For experiment #1, an ameroid constrictor (Research Instruments SW, Escondido, CA, USA) with an inner diameter of 0.5 mm was placed around the right common carotid artery (CCA) and a microcoil (Wuxi Samini Spring Co., Ltd., Wuxi, China) with an inner diameter of 0.18 mm was wrapped around the left CCA of C57BL6/J mice (C57VCI). By the sacrifice time point, the ameroid constrictor (inner diameter: 0.5 mm) reached near occlusion (Figure 9A, arrowhead). For experiment #2, the previously reported bilateral placement of ameroid constrictors [35] was applied to the 5xFAD mice to induce VCI (5xVCI). Ameroid constrictors (Research Instruments SW, Escondido, CA, USA) with an inner diameter of 0.75 mm were bilaterally applied to the CCAs (Figure 9B). At the time of sacrifice, swelling of casein (inner material of ameroid constrictors) was evident but the opening was not fully occluded (Figure 9B).

### 4.4. Culture and Preparation of Human MSCs

Human MSCs that were isolated from the Wharton’s Jelly were used for this study [23]. MSCs were cultured in minimal essential alpha 1x medium (MEMα1x; Gibco, Waltham, MA, USA) that contained 10% fetal bovine serum (FBS; Biowest, Riverside, MO, USA) and 0.5% gentamicin (Gibco, Waltham, MA, USA) at 37 °C in a 5% CO_2_ incubator. Passage 6 MSCs were expanded and cultured in a 175T flask (Thermo Fisher Scientific, Waltham, MA, USA) until confluency reached 80–90%. The surface markers used to characterize the human MSCs via fluorescence-activated cell sorting (FACS) are illustrated in Figure S2. Immunophenotyping was carried out by referring to previously reported procedures [56,57]. The MSCs used in this present study met the criterion set by the International Society for Cellular Therapy (ISCT) to define MSCs [58]. To prepare the cells for injection, MSCs were first washed with Dulbecco’s phosphate-buffered saline (DPBS; Biowest, Riverside, MO, USA) and then detached from the flasks using 0.25% Trypsin-Ethylenediaminetetraacetic acid(EDTA) (Gibco, Waltham, MA, USA). MSCs were suspended in serum-free phenol red-free MEMα1x (Gibco, Waltham, MA, USA) at concentrations of 1 × 10^5^/ 2 µL [40] and 1 × 10^5^/ 5 µL [41] for experiments #1 and #2, respectively (Figure 9).

### 4.5. Transplantation of MSCs into the Lateral Ventricle of Mice

While maintaining anesthesia at 1.5–2% of isoflurane (Hana Pharmaceutical Co., Ltd., Seoul, Korea), MSC transplantation was carried out by using a stereotaxic frame (Harvard apparatus, Holliston, MA, USA). MSCs were injected bilaterally to the lateral ventricles of mice at the following coordinates: −0.4 mm posterior to bregma, ±1.0 mm from the midline, and 2.3 mm ventral from the surface of the skull (Figure 9). A 25-µL Hamilton syringe (Hamilton Company, Reno, NV, USA) was used to complete the injection. To prevent backflow, there was a 5-min delay before slowly withdrawing the syringe. For experiment #1, an injection of 2 µL of MSCs (1 × 10^5^) or vehicle (phenol red free MEMα1x) were made at a rate of 0.5 µL/min. Mice received a total of 4 µL of MSCs or vehicle. For experiment #2, an injection of 5 µL of MSCs (1 × 10^5^) or vehicle (phenol red free MEMα1x) at a rate of 0.5 µL per min was given. Mice received a total of 10 µL of MSCs or vehicle.

### 4.6. Behavior

To assess alterations in spatial working memory and motor coordination, the Y-maze and rotarod tests were conducted, respectively. Behavioral performances were examined twice, before (experiment#1: 4 days after VCI, experiment #2: 18 days after VCI) and after administration of MEM or MSCs (experiment #1: 6 days after injection, experiment #2: 26 days after injection) (Figure 9). Procedures reported from a previous study [15] were applied to conduct the Y-maze test and calculate the spontaneous alternation performance (SAP)%. By referring to a previously reported protocol [59], mice were placed on a rotating rod and were tested for 3 trials each day for 3 consecutive days. Before each trial, mice were trained for a minute on the rotating rod at a speed of 4 rpm. For the actual trial, the speed of the rotating rod was accelerated from 4 to 40 rpm for a total of 5 min and during that period the latency to fall (s) was recorded. There was a 10-min recovery time in between each trial.

### 4.7. MR Imaging and Fiber Tractography

T2 weighted spin echo magnetic resonance (MR) images were acquired by using a 7.0T Bruker Biospin instrument (Bruker-Biospin, Ettlingen, Germany). The acquisition parameters were optimized from a previous study [15]: Repetition time (TR)/echo time (TE) = 3000/60 ms, slice thickness = 0.5 mm, echo train length = 4; in-plane resolution = 100 × 100 μm^2^; and number of averages = 6. Subsequently, diffusion weighted spin echo images were also acquired using the following parameters: TR/TE = 2000/30 ms, slice thickness = 0.5 mm, number of slices = 16, gradient direction = 30, diffusion gradient duration = 4.5 ms, diffusion gradient separations = 10.6 ms, and b-values = 1000 s/mm^2^, number of average = 4, field of view (FOV) = 20 × 15 mm^2^, matrix = 128 × 96, and in-plane resolution = 156 × 156 μm^2^. To examine alterations in the white matter structure, specifically the corpus callosum, diffusion tensor imaging (DTI) tractography was analyzed by using the Diffusion Toolkit and TrackVis software (www.trackvis.org). As reported previously, normalized fractional anisotropy (FA) and tract density (TD) values were quantitated by dividing the FA or TD of the corpus callosum by the FA or TD of the whole brains of the mice [15].

### 4.8. Immunohistochemistry

At the respective sacrifice time points, mice were sacrificed via cardiac perfusion. Brain tissues were harvested and fixated in 4% paraformaldehyde (PFA; Biosesang, Seongnam, Korea) prior to making paraffin blocks. Coronal sections (thickness: 4 µm) of the blocks were made by using a microtome (Leica Biosystems, Wetzlar, Germany). To initially assess for structural alterations, hematoxylin & eosin (H&E) was carried out and examined first. Immunohistochemical (IHC) staining was conducted as reported previously [15,60]. The following primary antibodies were used: Ionized calcium binding adaptor molecule 1 (Iba-1, 1:250; Wako Chemicals, Richmond, VA, USA), neuronal nuclear antigen (NeuN, 1:400; Millipore, Temecula, CA, USA), leukocyte common antigen (CD45, 1:200; Biolegend, San Diego, CA, USA), human cytoplasmic protein (STEM121, 1:500; Cellartis–Takara Bio, Kusatsu, Japan), and the amino acid residue 1–16 of beta amyloid (Aβ) (6E10; 1:250; Biolegend, San Diego, CA, USA). The secondary antibodies were as follows: Alexa Fluor 546-conjugated goat anti-mouse (1:400), Alexa Fluor 546-conjugated donkey anti-rabbit (1:400), Alexa Fluor 633-conjugated donkey anti-rat (1:400; Life Technologies, Carlsbad, CA, USA), and Dako EnVision + System-HRP Labelled Polymer anti-mouse (Dako, Carpinteria, CA, USA).

Coverslip mounted slides were scanned by using a confocal microscope (Carl Zeiss AG, Jena, Germany). The ImageJ image processing program (National Institutes of Health (NIH)) was utilized to analyze the percentage of NeuN and STEM121-positive cells. For the Aβ (6E10)-stained slides that underwent 3,3′-Diaminobenzidine (DAB; Dako, Carpinteria, CA, USA) staining, slides were scanned by using a Scanscope AT scanner (Leica Biosystems, Wetzlar, Germany). The area percentage of Aβ burden and percentage of CD45 and Iba-1-positive cells were quantitated by using the InForm 2.4.1 image analysis software after acquiring images using the Vectra^®^ Automated Imaging System (version 2.4.1, PerkinElmer Applied Biosystems, Waltham, MA, USA).

### 4.9. Data Analysis

Statistical analysis was carried out using the GraphPad Prism 8.0 software (version 8, GraphPad, San Diego, CA, USA). All results are presented as mean ± standard error of mean (S.E.M). A student’s *t*-test (unpaired, two-tailed) was used to assess significance and a *p*-value ≤ 0.05 was considered statistically significant. The Kaplan Meier survival curves were also drawn by using the GraphPad software.

## 5. Conclusions

Collectively, the results of this present study suggest that first, the intracerebroventricular route is a feasible route to deliver MSCs into the brains of mouse models where VCI was induced via the combination of ameroid constrictors and microcoils or ameroid constrictors alone. Second, depending on the method used to induce chronic hypoperfusion, there will be a wide variation on the pathological disease progression, which will affect the survival rate of the mice differently.

## Supplementary Materials

Supplementary materials can be found at https://www.mdpi.com/1422-0067/21/15/5524/s1. Figure S1. Determining the origin of the hypointense signals from the hippocampal fimbria. L indicates left and R indicates the right hemisphere of the mouse brain. The location of each the coronal MR sections is illustrated by the sagittal section of the mouse brain (top): (1) forceps minor of corpus callosum (+1.70 mm anterior to bregma), (2) external capsule of the corpus callosum (+0.62 mm anterior to bregma), (3) the injection point (lateral ventricles) and the caudate putamen can be visualized (−0.22 mm posterior to bregma), and (4) the hippocampal fimbria and hippocampus can be detected (−1.82 mm posterior to bregma). A representative T2 weighted image from the MSC group where hypointense signals were observed from the hippocampal fimbria is shown on the left (section #3). When MR images past the lateral ventricles (−1.82 mm posterior to bregma) was observed (section #4), the presence of vertical streaks (red dotted lines) penetrating up to the ventral region of the hippocampus as hypointense signals is shown on the right. When H&E staining was performed from the equivalent section were signals were detected in the MR images, damage to the cortex and a vertical needle track is identified (region demarcated by a white, dotted line). Scale bar = 2 mm (whole brain). Figure S2. Immunophenotypic characterization of human mesenchymal stem cells via flow cytometry. Human MSCs were stained with isotype-matched monoclonal antibodies (gray shaded histogram) or with antibodies against specific surface markers (pink shaded histogram). The expressions of positive surface markers (CD105, CD73, CD90, and CD166; all above 95%) are shown on the top row and the expressions of negative surface markers (CD45, CD34, CD14, CD19, and HLA-DR; all below 2%) are illustrated in the bottom row.
