# Supplementary material for: Exploring the Potential of Mesenchymal Stem Cell-Based Therapy in Mouse Models of Vascular Cognitive Impairment
Source: Int J Mol Sci. 2020 Aug 1;21(15):5524. doi: 10.3390/ijms21155524 (PMC7432487; doi:10.3390/ijms21155524)
Supplement: Supplementary file 1 [file ijms-21-05524-s001.docx]

Supplementary Materials


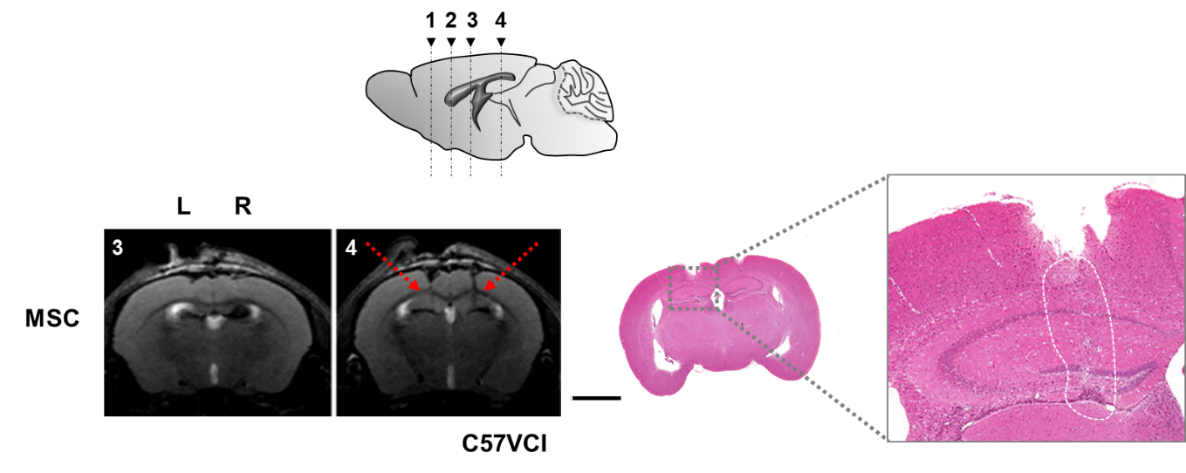


**Figure S1.** Determining the origin of the hypointense signals from the hippocampal fimbria. L indicates left and R indicates the right hemisphere of the mouse brain. The location of each the coronal MR sections is illustrated by the sagittal section of the mouse brain (top): (1) forceps minor of corpus callosum (+1.70 mm anterior to bregma), (2) external capsule of the corpus callosum (+0.62 mm anterior to bregma), (3) the injection point (lateral ventricles) and the caudate putamen can be visualized (-0.22 mm posterior to bregma), and (4) the hippocampal fimbria and hippocampus can be detected (-1.82 mm posterior to bregma). A representative T2 weighted image from the MSC group where hypointense signals were observed from the hippocampal fimbria is shown on the left (section #3). When MR images past the lateral ventricles (-1.82 mm posterior to bregma) was observed (section #4), the presence of vertical streaks (red dotted lines) penetrating up to the ventral region of the hippocampus as hypointense signals is shown on the right. When H&E staining was performed from the equivalent section were signals were detected in the MR images, damage to the cortex and a vertical needle track is identified (region demarcated by a white, dotted line). Scale bar = 2 mm (whole brain).


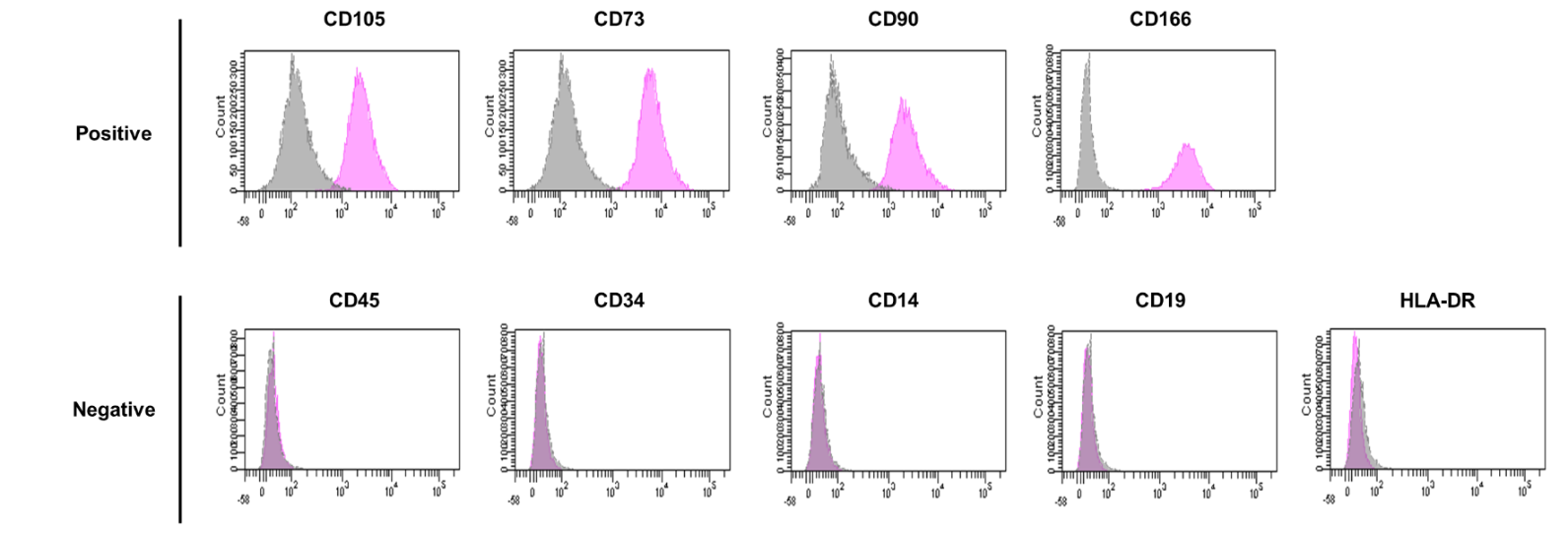


**Figure S2.** Immunophenotypic characterization of human mesenchymal stem cells via flow cytometry. Human MSCs were stained with isotype-matched monoclonal antibodies (gray shaded histogram) or with antibodies against specific surface markers (pink shaded histogram). The expressions of positive surface markers (CD105, CD73, CD90, and CD166; all above 95%) are shown on the top row and the expressions of negative surface markers (CD45, CD34, CD14, CD19, and HLA-DR; all below 2%) are illustrated in the bottom row.
